# Mr. Dunning, on Inoculation

**Published:** 1805-07-01

**Authors:** Richard Dunning

**Affiliations:** Dock


### *To the Editors of the Medical and Physical Journal.*

GENTLEMEN,

ALWAYS vaccinists from principle, and now from a conviction, which has arisen out of very extensive opportunities for observation, many of the medical practitioners in in these towns will never hesitate to publish from time to time the results of their experience in the practice of vaccination, whatever these may be; indeed, were they sometimes, for instance, on occasions like the present, to neglect such communications, they would certainly ill discharge those duties which attach to the profession, and which, therefore, the public have a right to expect from them; for these reasons, I hope you will give a place in your next Journal to the following letters, with which I have been just favoured from three highly respectable surgeons resident in this town and Stonehouse, which immediately adjoins it. I saw the cases which they record; connected with the appearances on Master Rendall's arm, which are faithfully detailed in my minutes; with some of Mr. Goldson's cases; with some of the experiments instituted at the Vaccine Institution; with those of Dr. Rollo; with the cases of the Rev. Mr. Hitchins's child; Mr. Wallis's; and with some others of a similar description, which, if necessary, can be adduced. These communications incontrovertibly establish, I think, the modifying power of the vaccine over the variolous principle, where the former has been inadequately or imperfectly impressed. This idea, which early struck me, was for some time entirely resisted by many medical men here, who now most unequivocally subscribe to it. The question then is, whether this imperfect action of the vaccine virus arise from circumstances previous to or adventitious during the process of vaccination, or *necessarily from a high variolous susceptibility, which a single vaccination, (complete in all its characters as far as we can ascertain them) cannot entirely subdue that susceptibility,* which, when rouzed into action by casual exposure, invariably and rapidly sweeps away the miserable objects of it, and which, when excited by art, always subjects to the severest disease, very often mutilates, and very often destroys.

A fruitful source of *incomplete vaccination,* we are taught by the highest authority, will be found in herpetic eruption. Speaking of herpes in the last Journal, Dr. Jenner observes, that this establishes every gradation of *vaccine insusceptibility,* from that which subjects to no risk of future variolous impression to that which denies all security against it, or becomes what I have termed spurious vaccination. Dr. Jenner's words are, "During the presence of herpes there may be no imperfection in the vaccine pock, and there may be, and there certainly is, every gradation between that slight deviation which is of no consequence, up up to that which completely denies all security." Another source of incomplete vaccination at least is found in the use of too early or too late fluid; taken any considerable time on either side of the acme of the vesicle, this must be less active or less perfect; for, as I have elsewhere said on the same subject, "sunt certi denique fines, quos ultra citraque nequit consistere rectum." The coincidence of the broken vesicle, with every case of secondary variolæ which have hitherto fallen under my own observation, has been so uniform and remarkable, that it justifies, I think, (at least so long as we are at a loss for other more satisfactory causes of *incomplete vaccination, I do not say of failures)* all the importance which has been deduced from this circumstance. The following cases, and that given us by Mr. Forbes in a very excellent and discriminative paper in the last Journal, for which I beg him to accept my thanks, tend directly to countenance a belief that we have in this source a very frequent cause at least of secondary variolæ. Stephen Brown was infected in one arm only, and John Heath was vaccinated from this arm; the small-pox which occurred in Brown were of the mildest kind, and very early reached maturation; on February 15, red spots only appeared, on the 19th, matter was taken from them for inoculation ?

Quere. Were not these secondary variola or variolæ modified by previous inoculation.

Aboriginal variolous insusceptibility at the time of vaccination, (under which condition of the system there is much reason to believe that vaccinæ, perfect or nearly perfect in all their characters, can sometimes be produced and regularly transmitted to other subjects) will in all probability be now and then a source of *regular small-pox, or small-pox of every description* subsequent to vaccination. Here the anti-vaccinist exultingly tells us, that we have neither constitutional affection nor eruption, that is, *neither fever nor abscess,* to assure us when our object has been obtained; that the absence of these desirable concomitants in subjects of the inoculated small-pox always places them on their guard. Granted, it may be a little deficit, we leave him in the enjoyment of this inconsiderable and exclusive advantage, and heartily congratulate ourselves, and our little patients, on their exemption from those dreadful accessories.

Casual observation, and the results of some experiments purposely undertaken, will enable me I hope, by and by, to offer some minutes and remarks on the different influences of of the vaccine principle on subjects of different variolous insusceptibilities; these insusceptibilities are derived to the human body from different sources; from inoculated small-pox, from vaccination, from high degrees of herpes, of psora, and, perhaps, from some other conditions of the skin; there exist also, constitutionally, temporary and permanent insusceptibilities; each of these insusceptibilities, I have reason to believe, prevents a specific excitability or modification relatively to the vaccine principle. Such enquiries as these are apparently minutiæ, and of course the cui bono of them will not be evident to all; those however who think with me, that we are yet perhaps in limine, with respect to the physiology of the skin, and exanthematous affections, will venture to believe that these and similar investigations may be prosecuted with ultimate usefulness.

Notwithstanding our united and zealous exertions in the cause of vaccination, I grieve to add that not less than one hundred and fifty adults and children in this town only, Dock, (I do not include Plymouth and Stonehouse) have been destroyed by the small-pox within the present year; *fifty were destroyed by it in May,* and its victims continue to die daily in the same proportion; these melancholy events we believe have entirely followed the introduction of this spotted pestilence among us by the inoculations of an old superannuated common labourer. This man, I am told, armed with a thousand horrid tales of cow-pock mischief, first literally scares or conciliates the timid and the prejudiced, and then, with matchless impudence and unfeeling, introduces the deleterious poison into their families, and thus spreads it in every direction. Shocked the more at this dreadful destruction of human life, because convinced that nearly the whole of it might have been, and that repetitions of it can be, prevented, I ask with every possible respect, but with the most anxious solicitude, whether this subject be wholly unbecoming the dignity of some legislative or magisterial enquiry? While we hear with vast satisfaction the small-pox have been nearly exterminated from Berlin, Vienna, and some parts of France, we must lament that we have been out-stripped here in the great cause of humanity. I make the following extract from a letter replete with acute remark, highly favourable to the interests of vaccination, with which I was favoured three days ago by Mr. Bryant, surgeon at Callington, whose extensive practice has afforded him an opportunity of seeing a great deal of both inoculations: "About three years since I was applied to on a sudden emergency to inoculate about forty people, of all ages, on the breaking out of the small-pox in their neighbourhood; not having variolous matter which I could depend on, I vaccinated the whole, and three days after having procured efficacious means, I inoculated all with small-pox whom I judged had not been certainly infected with the vaccine lymph. About twelve of these were infected with cow-pock in one arm, and small-pock in the other; the progress of each seemed to go on perfectly well, without the least effect on one another, to the point of eruption, the variolous arm shewing marks of eruption at the usual period though three days later than the other; but neither patient was disordered, nor had either an eruption; though those who had failed taking the vaccine infection, and consequently had the small-pox alone, had the usual symptoms and eruptions, but not many." I am, &c.

*Dock, June* 13, 1805. RICHARD DUNNING.

---

"To Mr. DUNNING, Surgeon.

"Dear Sir,

"KNOWING no medical man in this neighbourhood, who has evinced a greater degree of laudable zeal for the introduction and propagation of vaccination than yourself, nor any person who has laboured more diligently to impress on the minds of the inhabitants of this place, a due sense of its importance; thinking also, as you do, that every case of failure, in place of being concealed ought to be openly avowed and acknowledged, I feel no hesitation in communicating to you a brief history of an instance of that kind which lately occurred to me in these Barracks. How far the nature of the case may correspond with some of your late ingenious conjectures, I must leave you to judge.

"About two years and a half ago, I inoculated the male child of a Sergeant Ward in both arms with fresh vaccine virus, taken at the time from the arm of another child. The infant inoculated was then about six weeks old; this first inoculation failed, as did also a second and a third. By much persuasion the mother at length allowed me to make a fourth trial, which was done, as in the former instances, with fresh matter. I saw no more of the child until the seventh day after inoculation. On inspecting the arms, the virus appeared to have produced no visible effect on the right, but on the left completely so. *The apex of the vesicle however appeared to have been broke,* and the the mother informed me, that in the morning she found *the shirt adhering to it slightly.* The areola was about half formed, I mean as to its greatest diameter, for it spread afterwards to about the breadth of a half crown piece. I saw the child frequently afterwards, and as every appearance exhibited the characteristic mask of genuine vaccination, I confess, I considered him as perfectly secure. I was however not a little surprized about the middle of last month to hear, that this same boy laboured under very benign and distinct small-pox. As both you, and our ingenious friend and neighbour Mr. Little, saw the child both before and after I was acquainted with the circumstance, it is unnecessary for me to make any observations on the nature of the disease. I believe you were both agreed, that the child laboured under general variolæ, but certainly of an irregular kind; because the pustules were in a perfect state of maturation on the fifth day after the first appearance of eruption, and completely desiccated by the eighth. There is only one other circumstance which I wish to mention relative to this child, and that is, that on examining the left arm the foveola or indentation, always I believe observable after perfect vaccination, is in this subject extremely small. This, and similar cases, will not at all lessen my confidence in vaccination.

"I am, &c.

"JOHN MAGIN,

"Surgeon, Plymouth Division of Royal Marines.

*Royal Marine Infirmary, May* 22, 1805."

In reading this case, every person will be struck with the vaccine insusceptibility of the subject of it; the cicatrix is indeed so slight that even repeated and accurate examinations of the arm are almost necessary to discover it.

R. D.

---

"To Mr. DUNNING.

"Dear Sir,

"CONNECTED with the cases of secondary small-pox is the following, which I beg to give you. On May 17, 1800, I vaccinated the daughter of Mr. Luscombe, then about five months old; it took in one arm only, and the character of the vesicle was thought perfect, so as to produce the necessary constitutional impression and consequent protection. She has been twice since intentionally exposed to small-pox contagion, which was resisted in each instance.

stance. In the beginning of this month (May) she was again exposed to a highly variolated atmosphere, wherein a child lay ill of that disease, and *in a few days after was attacked with fever and sickness;* this left her on the same evening; she remained well the two following days, when fever commenced, and a few eruptions followed on the face, trunk, and extremities, in number about forty or fifty; they continued four, and a few of them five days, when they completely and almost suddenly desiccated; an appearance of a peculiar kind was remarked, surrounding some of the largest of these eruptions on the body as they went off; that of a *distinct fringed circle,* rather more than half an inch from the centre of the pustule, (but unaccompanied with an inflamed base) so much resembling that which often defines a well marked vaccine vesicle just beginning to decline; this is a peculiarity which I have never seen in any eruptive disease except the vaccine (some varieties of tetter more resemble what I mean).

This appearance, which forcibly strikes me, and which I believe is an occurrence *depending upon previous vaccine impression not having completely pervaded the system, but yet enough to modify the small-pox, and totally disarm it of all its malignity,* is of too much importance to be passed over without particular notice, and ought to excite our nicest attention to the state of the skin in particular, which we now know so well from experience is such as often to derange the due action of the vaccine virus, when least suspected. When we are also aware of the very nice laws by which its action is impressed in very many instances, so as to elude our nicest observations, and yet permanent security obtained, we must not feel disappointed at the very few cases of *secondary small-pox* which we have seen, but follow up with increased ardour and increased observation this invaluable discovery. Let us do this, and I feel persuaded, nay almost convinced, that further time and proper observation will enable us by and by to explain the causes of, and the anomalies met with in, those constitutions, on which the partial failure depends. What I have remarked, appears to me, to apply to all the cases of *secondary small-pox* which I have seen after *incomplete vaccination;* that there *is a chain of peculiar modification,* by which they have been all characterized, whether they have been from *casual infection or inoculation,* bespeaking *the identity of the exciting vaccine principle partially obtained.* This child having been vaccinated among some of the earliest subjects in this place, I am not prepared from memory mory to say if any thing particular occurred at the time; whether the pustule might have been broken, or matter taken from it for other inoculations; these were circumstances, at that time, not so well attended to as they deserve.

I am, &c.

*Dock, May* 29, 1805. "D. LITTLE"

I never saw the effects of broken vesicle more strongly marked than on this child's arm, and Mr. Little has said he cannot doubt his having opened it. R. D

---

"To Mr. DUNNING.

"Dear Sir,

"UNDERSTANDING that you are about to publish a few instances of variolous eruptions which have taken place in this town after inoculation for the cow-pox, I am induced to send you the history of a case of this kind, which has lately occurred in my practice, which, from particular circumstances, appears to me to admit of some explanation, and not to militate against the practice of vaccination. If you consider it worthy of attention, it is at your service to dispose of it as you think proper. About four years since I vaccinated the fourth child of Mr. Bryant, in King Street, then about seven weeks old; she received the infection perfectly on one arm, but the operation failed on the other; at an early period of the vesicle, the eighth day or succeeding day or two, I took the fluid freely for inoculation, breaking down the vesicle, and allowing as much to escape as I could obtain for my purpose at every application of the lancet. I know of no remarkable circumstances attending the future progress of the arm during the desiccation and cure. On the 10th of last month (May) I was called to visit this child, to see some eruptions which were then making their appearance, and were suspected to be small-pox. I did not hesitate to say immediately that I thought them small-pox; but from the distinctness of them, confided in their going on with perfect safety to my little patient, although she was tolerably well sprinkled. On the following day I was confirmed in my prognostick, that they were mild, and certainly felt justified in declaring it as my opinion that they were *peculiarly affected by the previous inoculation* (the vaccine); they might fairly be called *vaccinated variolæ,* by which I mean variolæ produced under the influence of a certain vaccine state of the constitution, constitution, and which it appears to me probable, had reduced both the rise and progress of the eruption to a state vastly more favourable than is usually the case where no such previous affection has existed. I wish to remark a very striking appearance during the inflammatory stage of these eruptions, as the reason *for my laying emphasis on the previous effect of the vaccine inoculation* on the habit. It is, that the areola surrounding them was to a much greater extent than the redness seen to surround small-pox in the ordinary way, and if viewed at a little distance they appeared more like small *vaccine vesicles* than genuine variolæ; only, of course, not so flattened at the apex. In the cicatrix or mark remaining at the place of inoculation, instead of the usual appearances of an even surface, excepting some small and rather regular puncta, such as may be seen on the rind of a lemon, or nearly like them, *there is a seam,* such as is observed *after the healing of a part which has been scalded and healed irregularly.* This I take to be *owing to the violence done to the vesicle while breaking it down for the purpose of extracting the contained fluid.* The result of this case has not destroyed the confidence of the parents in the preservative power of cow-pox, for about twenty days since I vaccinated another infant in the same family, who has gone through the inoculation satisfactorily, though it only took effect in one arm; and I now hope the infection of small-pox has not previously taken place; the child has not been kept from its sister, nor have two others who were vaccinated some years since; they resist the small-pox. This case, as well as others attended with *similar circumstances and consequences,* strengthen my confidence in the propriety of vaccine inoculation, *for they are all instances of infection in one arm only where the vesicles have been broken down early, and the fluid consequently allowed to escape, and have been generally coupled with other subjects in the same families who resist small-pox subsequently to being inoculated with cow-pox, although exposed to its influence at the same time, and in an equal degree.* Future experience must determine, whether the breaking down a single vesicle at an improper period (which I must at present believe to be the most general impediment); some idiosyncrasy of the constitution, which regulates the susceptibility; or what other causes, interfere to prevent in individual cases *complete saturation;* there may be many; these I leave to abler practitioners to determine, of what number and description, while I cannot help help expressing my warmest hope that such investigation will be attended with every success, and ultimately tend to the general adoption of the Jennerian practice.

I am, &c.

*Dock, June* 9, 1805. JOHN J. SMITH.

